# A guide to selecting high-performing renewable antibodies for KIF5A (Q12840) across western blot, immunoprecipitation, and immunofluorescence

**DOI:** 10.12688/f1000research.184178.1

**Published:** 2026-06-30

**Authors:** Riham Ayoubi, Charles Alende, Maryam Fothouhi, Sara González Bolívar, Vincent Francis, Peter S. McPherson, Carl Laflamme, NeuroSGC/YCharOS/EDDU collaborative group

**Affiliations:** 1 Department of Neurology and Neurosurgery, Structural Genomics Consortium, The Montreal Neurological Institute, McGill University, Montreal, Canada

**Keywords:** UniProt ID Q12840, KIF5A, KIF5A, Kinesin Family Member 5A, Kinesin heavy chain isoform 5A, antibody characterization, antibody validation, western blot, immunoprecipitation, immunofluorescence

## Abstract

Kinesin Family Member 5A (KIF5A) is an important protein for anterograde cellular transport. We characterized ten commercially available research antibodies against KIF5A for use in western blot, immunoprecipitation, and immunofluorescence using a standardized workflow. Antibody performance was assessed by comparing signal in a wild-type cell line and its corresponding knockout derivative. This work is part of a broader collaborative public-good initiative to improve biomedical research by systematically evaluating commercial antibodies against human proteins and openly sharing the data as a resource for the scientific community. We encourage readers to use this report as a guide for selecting antibodies best suited to their specific applications.

## Introduction

KIF5A is a member of the KIF5 family of kinesin motor proteins that mediates anterograde transport along microtubules. In neurons, it plays a critical role in the transport of mitochondria and synaptic vesicles from the soma to the synapse, supporting axonal maintenance and function. ^1^ Mutations in the *KIF5A* gene impair cargo transport and are associated with amyotrophic lateral sclerosis (ALS). ^2^

Here, we characterized ten antibodies against KIF5A, for use in western blot, immunoprecipitation, and immunofluorescence using standardized protocols. Antibody performance was assessed by comparing signal in wild-type (WT) and knockout (KO) cell lines. The resulting data can guide the selection of antibodies best suited to specific research needs, enabling robust biochemical and cellular assessment of the target.

This work is part of a broader collaborative initiative involving academics, funders, and antibody manufacturers, aimed at improving biomedical research reproducibility through the systematic characterization of commercial antibodies against human proteins and open data sharing. Antibodies are provided in-kind by participating manufacturers. The approach involves identifying cell lines with sufficient target expression, generating or sourcing corresponding KO cell lines, and evaluating commercially available antibodies, with a focus on renewable (monoclonal and recombinant) reagents. ^3^ We do not provide explicit antibody recommendations; rather, the data are presented to enable independent interpretation. Guidance on data interpretation is available via the YCharOS gateway ^4^ and in Table 4 of this data note.

**Table 1.** Summary of the cell lines used.

Institution	Catalog number	RRID (Cellosaurus)	Cell line	Genotype
Abcam	ab278079	CVCL_0022	U-87 MG	WT
Abcam	ab306717	CVCL_F4C9	U-87 MG	*KIF5A* KO

**Table 2.** Summary of the KIF5A antibodies tested.

Company	Catalog number	Lot number	RRID (Antibody Registry)	Clonality	Clone ID	Host	Concentration (μg/μL)	Vendors recommended applications
Abcam	ab322900 **	1113400–1	AB_3698263	recombinant mono	EPR29540–31	rabbit	0.507	Wb, IF
Abcam	ab5628	1081534–6	AB_2132218	polyclonal	-	rabbit	1.000	Wb, IP, IF
Aviva Systems Biology	ARP33868_P050	QC3274	AB_841511	polyclonal	-	rabbit	0.500	Wb
Cell Signaling Technology	92872 **	1	AB_3698333	recombinant mono	F2W1W	rabbit	0.000	Wb
GeneTex	GTX104875	39708	AB_1241009	polyclonal	-	rabbit	1.000	Wb
GeneTex	GTX113761	40142	AB_2037309	polyclonal	-	rabbit	0.230	Wb
Proteintech	21186–1-AP	14632	AB_10733125	polyclonal	-	rabbit	0.287	Wb, IP, IF
Proteintech	67009–1-Ig *	10008940	AB_2882326	monoclonal	1A7E3	mouse	1.000	Wb,
Proteintech	84013–4-RR **	23010610	AB_3671581	recombinant mono	241124H1	rabbit	1.000	Wb, IF
Proteintech	84013–5-RR **	23011174	AB_3671582	recombinant mono	241124H5	rabbit	0.400	Wb

Wb = western blot; IF = immunofluorescence; IP = immunoprecipitation,

** recombinant antibody,

* monoclonal antibody

**Table 3.** Table of secondary antibodies used.

Company	Secondary antibody	Catalog number	RRID (Antibody Registry)	Clonality	Concentration (μg/μL)	Working concentration (μg/mL)
Proteintech	HRP-Goat Anti-Rabbit Antibody (H + L)	RGAR001	AB_3073505	recombinant polyclonal	1.0	0.05
Proteintech	HRP-Goat Anti-Mouse Antibody (H + L)	RGAM001	AB_3068333	recombinant polyclonal	1.0	0.5
Cell Signaling Technology	Protein A, HRP conjugate	12291	NA	polyclonal	0.125	0.5
Proteintech	CoraLite Plus 555-Goat Anti-Rabbit Antibody (H + L)	RGAR003	AB_3073507	recombinant polyclonal	0.5	0.5
Proteintech	CoraLite Plus 555-Goat Anti-Mouse Antibody (H + L)	RGAM003	AB_3068539	recombinant polyclonal	0.5	0.5

**Table 4.** Illustrations to assess antibody performance in all western blot, immunoprecipitation and immunofluorescence.

Western blot	Immunoprecipitation	Immunofluorescence
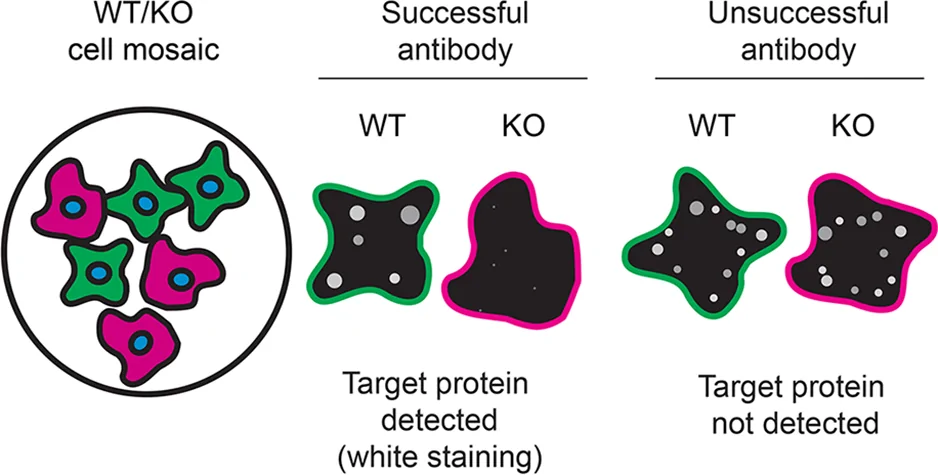	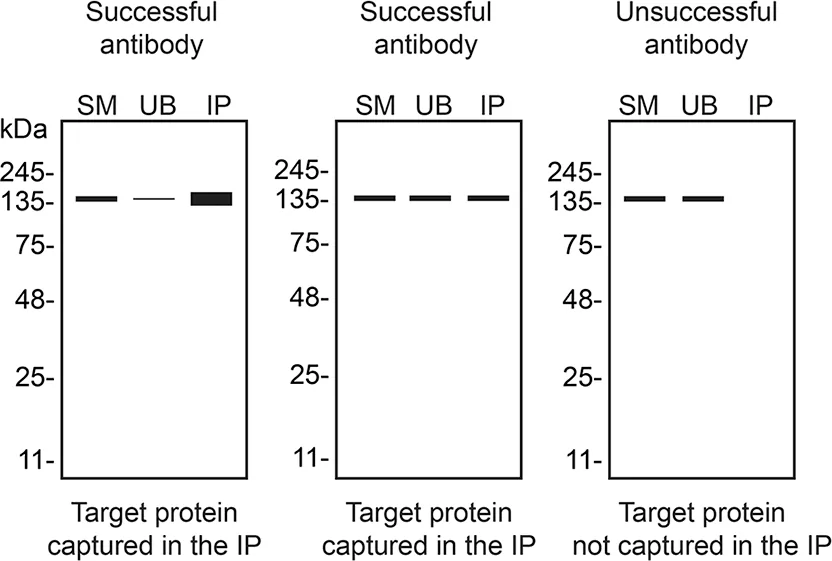	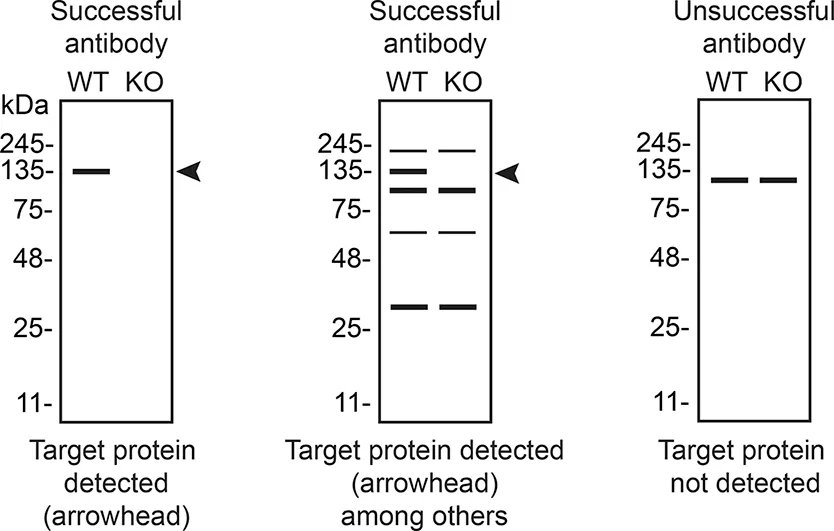

This table has been reproduced with permission from Ayoubi et al., Elife, 2023 ^5^

## Results and discussion

Our standard protocol involves comparing readouts from WT and KO cells. ^3^ The first step is to identify a cell line expressing sufficient levels of the target protein to generate a measurable signal with antibodies. To this end, we examined the DepMap transcriptomics database (Cancer Dependency Map Portal, RRID: SCR_017655) to identify cell lines with expression levels greater than 2.5 log2 (transcripts per million “TPM” + 1), a threshold we have found to be suitable. ^5^ The U-87 MG cell line expresses the *KIF5A* transcript at 4.1 log _2_ TPM + 1, and a corresponding U-87 MG *KIF5A* KO cell line was obtained from Abcam ( Table 1). Moreover, as seen on DepMap, the U-87 MG does not carry mutations in *KIF5A* that could affect antibody-epitope binding.

**Figure 1.**
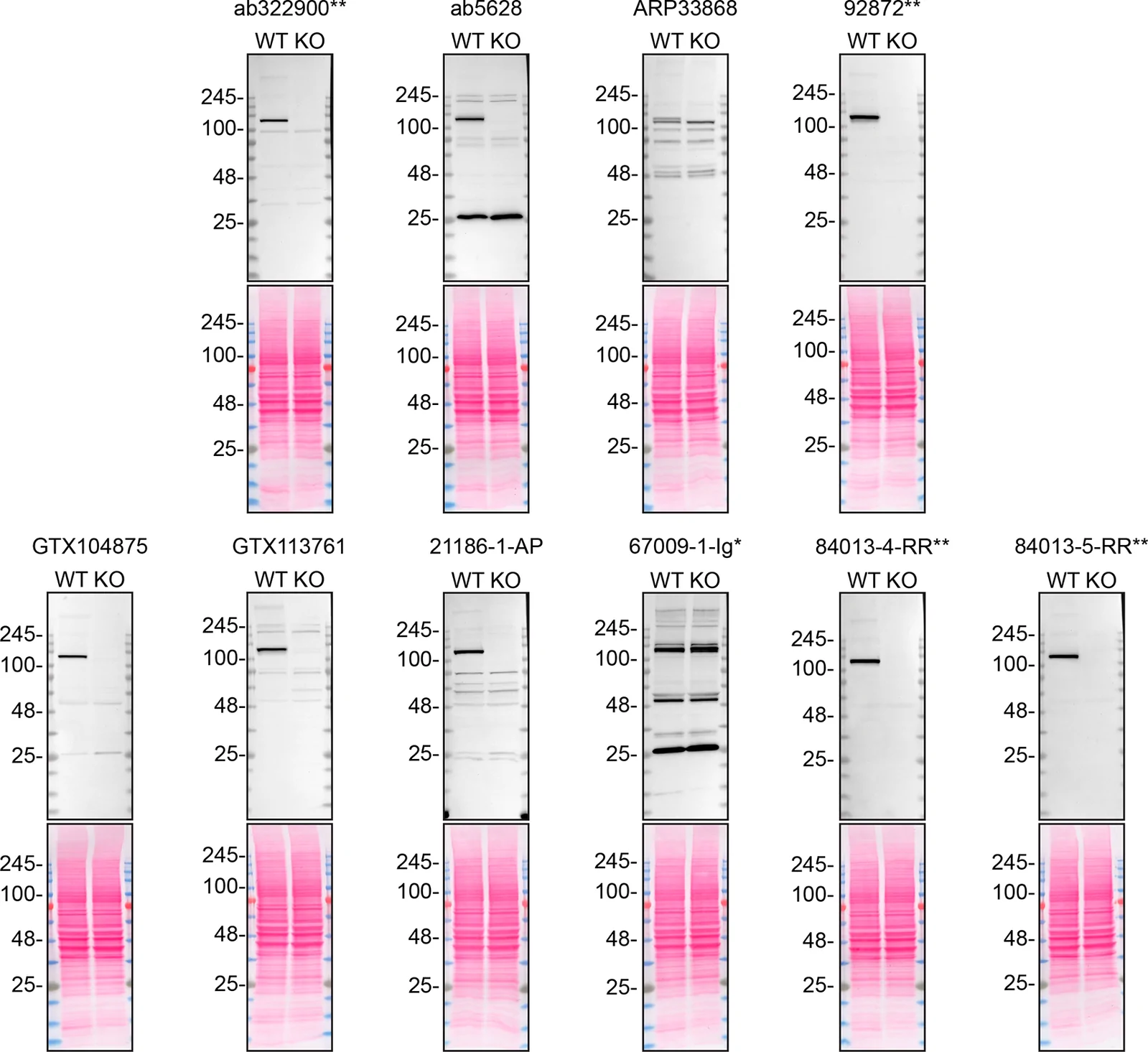
KIF5A antibody screening by western blot. Lysates of U-87 MG WT and *KIF5A* KO were prepared, and 60 μg of protein were processed for western blot with the indicated KIF5A antibodies. The Ponceau stained transfers of each blot are presented to show equal loading of WT and KO lysates and protein transfer efficiency from the acrylamide gels to the nitrocellulose membrane. Antibody dilutions were chosen according to the recommendations of the antibody supplier. Antibody dilutions used: ab322900** at 1/1000, ab5628 at 1/500, ARP33868_P050 at 1/500, 92872** at 1/500, GTX104875 at 1/500, GTX113761 at 1/500, 21186–1-AP at 1/500, 67009–1-Ig* at 1/500, 84013–4-RR** at 1/3000, 84013–5-RR** at 1/1000. Predicted band size: 117.3 kDa. ** = recombinant antibody, * = monoclonal antibody.

First, all ten antibodies were tested in western blot. WT and *KIF5A* KO protein lysates were resolved on SDS-PAGE, transferred to nitrocellulose membranes, and probed in parallel with KIF5A antibodies in parallel ( Figure 1).

**Figure 2.**
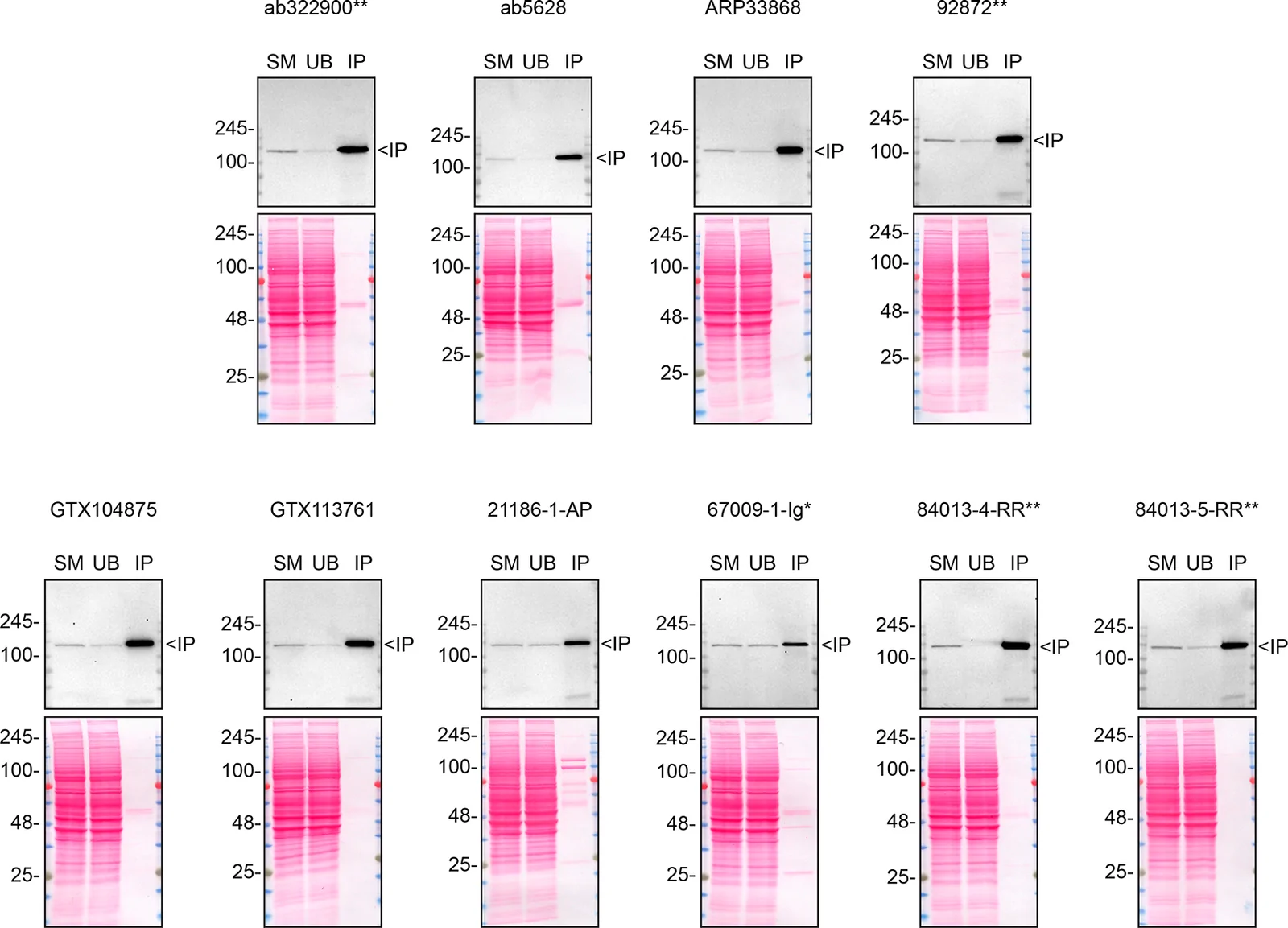
KIF5A antibody screening by immunoprecipitation. U-87 MG lysates were prepared, and immunoprecipitation was performed for 2 h using 3.0 mg of lysate and 2.0 μg of the indicated KIF5A antibodies pre-coupled to Dynabeads protein A or protein G. Samples were washed and processed for western blot with the KIF5A antibody 92872** used at 1/500. The Ponceau stained transfers of each blot are shown. SM = 2% starting material; UB = 2% unbound fraction; IP = immunoprecipitate. ** = recombinant antibody, * = monoclonal antibody.

The ability of antibodies to capture KIF5A from U-87 MG lysates was assessed by immunoprecipitation, followed by western blot analysis. Equal amounts of the starting material (SM) and the unbound fractions (UB), as well as the whole immunoprecipitate (IP) eluates were separated by SDS-PAGE. For detection, 92872**, a specific KIF5A antibody identified in Figure 1 was used ( Figure 2).

**Figure 3.**
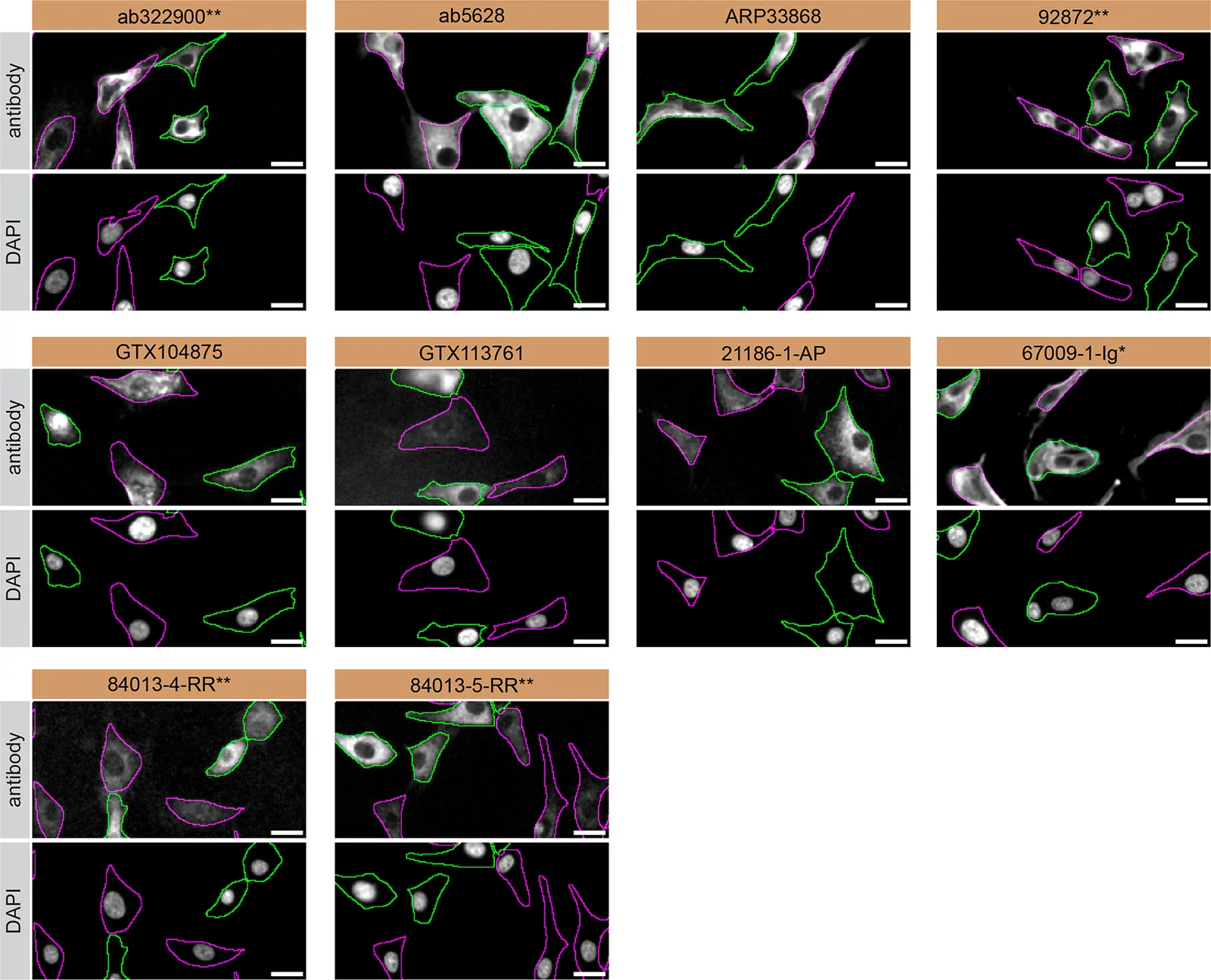
KIF5A antibody screening by immunofluorescence. U-87 MG WT and *KIF5A* KO cells were labelled with a green or a far-red fluorescent dye, respectively. WT and KO cells were mixed and plated to a 1:1 ratio in a 96-well plate with optically clear flat-bottom. Culture medium was removed, and cells were fixed with 4% PFA for 15 min at room temperature. Cells were permeabilized with 0.1% Triton-X100 for 10 min at room temperature and stained with the indicated KIF5A antibodies followed by the corresponding Fluor 555 coupled secondary antibody then with DAPI. Acquisition of the blue (nucleus-DAPI), green (WT), red (antibody staining) and far-red (KO) channels was performed. Representative images of the blue and red (grayscale) channels are shown. WT and KO cells are outlined with green and magenta dashed line, respectively. When an antibody was recommended for immunofluorescence by the supplier, we tested it at the recommended dilution and at 1 or 2 μg/ml. The rest of the antibodies were tested at 1 and 2 μg/ml, and the final concentration was selected based on the detection range of the microscope used and a quantitative analysis not shown here. Antibody dilutions used: ab322900** at 1/500, ab5628 at 1/1000, ARP33868_P050 at 1/250, 92872** at 1/1000, GTX104875 at 1/1000, GTX113761 at 1/200, 21186–1-AP at 1/200, 67009–1-Ig* at 1/1000, 84013–4-RR** at 1/250, 84013–5-RR** at 1/400. Bars = 10 μm. ** = recombinant antibody, * = monoclonal antibody.

For immunofluorescence, antibodies were screened using a mosaic strategy. WT and *KIF5A* KO cells were pre-labelled with distinct fluorescent dyes to enable discrimination between the two populations and imaged within the same field of view, thereby reducing staining, imaging, and analysis biais ( Figure 3). Quantification of immunofluorescence intensity across hundreds of WT and KO cells was performed for each antibody tested, ^1^ and representative images are shown in Figure 3.

In summary, we screened ten commercially available KIF5A antibodies in western blot, immunoprecipitation, and immunofluorescence by comparing the signal in WT and *KIF5A* KO cells. To assist with data interpretation, Table 4 outlines common antibody performance scenarios across applications. High-quality and renewable antibodies detecting KIF5A were identified for each application. Researchers studying KIF5A in other species are encouraged to consider these results and verify predicted species reactivity with the manufacturer before proceeding.

## Limitations

Inherent limitations are associated with the antibody characterization platform used in this study. First, the YCharOS project focuses on renewable (recombinant and monoclonal) antibodies and does not test all available antibodies against any given targets. While YCharOS partners provide access to the majority of renewable antibodies, some widely used polyclonal antibodies may not be included.

Second, the YCharOS approach is protein-agnostic and aims to provide objective data on antibody performance without predefined expectations regarding molecular weight or localization. As such, only a brief overview of the protein target is provided, and expert interpretation of banding patterns and subcellular localization is encouraged.

Third, experiments are not performed in biological replicates due to the parallel testing of multiple antibodies recognizing distinct epitopes. The identification of at least one specific antibody supports target expression in WT cells and its absence in KO cells, providing a reference for evaluating other antibodies. Experiments are performed using standardized conditions and master mixes to minimize variability. In immunofluorescence, testing at multiple antibody concentrations provides an additional assessment of specificity. Experiments may be repeated when no signal is detected. Furthermore, these data are generated independently of manufacturers’ validation processes, effectively constituting an external replication.

Finally, conclusions are limited to the experimental conditions and cell line used. The reliance on a single cell type represents a constraint, as protein expression levels can influence antibody performance. The use of cancer cell lines also introduces potential confounders, including mutations that may affect antibody-epitope binding. These limitations are inherent to cell line-based validation approaches.

## Method

The standardized protocols used for this antibody characterization study were established and approved by a collaborative group of academics, industry researchers, and antibody manufacturers. ^3^ Brief descriptions of the experimental procedures used in this study are described below.

### Cell lines and antibodies

The cell lines, primary and secondary antibodies used in this study are listed in Table 1, 2, and 3, respectively. To ensure consistency with manufacturer recommendations and account for proprietary formulations (where antibody concentrations are not disclosed), antibody usage is reported as dilution ratios rather than absolute concentrations. To facilitate proper citation and unambiguous identification, all cell lines and antibodies are referenced with their corresponding Research Resource Identifiers (RRIDs). ^6^ ^,^ ^7^ All cell lines used in this study were regularly tested for mycoplasma contamination and were confirmed to be mycoplasma-free.

### Antibody screening by western blot

U-87 MG WT and *KIF5A* KO cells were collected in RIPA buffer (25 mM Tris-HCl pH 7.6, 150 mM NaCl, 1% NP-40, 1% sodium deoxycholate, 0.1% SDS) (Thermo Fisher Scientific, cat. Number 89901) supplemented with 1x protease inhibitor cocktail mix (MilliporeSigma, cat. Number P8340). Lysates were sonicated briefly and incubated 30 min on ice. Lysates were spun at ~110,000 *x g* for 15 min at 4 °C and equal protein aliquots of the supernatants were analyzed by SDS-PAGE and western blot. BLUelf prestained protein ladder (GeneDireX, cat. Number PM008–0500) was used.

Western blots were performed with precast midi 4–20% Tris-Glycine polyacrylamide gels (Thermo Fisher Scientific, cat. Number WXP42012BOX) ran with Tris/Glycine/SDS buffer (Bio-Rad, cat. Number 1610772), loaded in Laemmli loading sample buffer (Thermo Fisher Scientific, cat. Number AAJ61337AD) and transferred on nitrocellulose membranes. Proteins on the blots were visualized with Ponceau S staining (Thermo Fisher Scientific, cat. Number BP103–10) which is scanned to show together with individual western blot. Blots were blocked with 5% milk for 1 hr, and antibodies were incubated O/N at 4 °C with 5% milk in TBS with 0,1% Tween 20 (TBST) (Cell Signalling Technology, cat. Number 9997). Following three washes with TBST, the peroxidase conjugated secondary antibody was incubated with the membrane in TBST with 5% milk for 1 hr at room temperature followed by three washes with TBST. Membranes were incubated with Pierce ECL (Thermo Fisher Scientific, cat. Number 32106) prior to detection with the iBright™ CL1500 Imaging System (Thermo Fisher Scientific, cat. Number A44240).

### Antibody screening by immunoprecipitation

Antibody-bead conjugates were prepared by adding 2 μg to 500 μl of Pierce IP Lysis Buffer from Thermo Fisher Scientific (cat. Number 87788) in a microcentrifuge tube, together with 30 μl of Dynabeads protein A- (for rabbit antibodies) or protein G- (for mouse antibodies) (Thermo Fisher Scientific, cat. Number 10002D and 10004D, respectively). All tubes were rocked for ~1 h at 4 °C followed by two washes to remove unbound antibodies.

U-87 MG WT were collected in Pierce IP buffer (25 mM Tris-HCl pH 7.4, 150 mM NaCl, 1 mM EDTA, 1% NP-40 and 5% glycerol) supplemented with protease inhibitor. Lysates were rocked 30 min at 4 °C and spun at 110,000 *x g* for 15 min at 4 °C. 1.5 ml aliquots at 2 mg/ml of lysate were incubated with an antibody-bead conjugate for ~1 h at 4 °C. The unbound fractions were collected, and beads were subsequently washed three times with 1.0 ml of IP buffer and processed for SDS-PAGE and western blot on precast midi 4–20% Tris-Glycine polyacrylamide gels.

### Antibody screening by immunofluorescence

U-87 MG WT and *KIF5A* KO cells were labelled with a green and a far-red fluorescence dye, respectively (Thermo Fisher Scientific, cat. Number C2925 and C34565). WT and KO cells were plated in a 96-well plate with optically clear flat-bottom (Perkin Elmer, cat. Number 6055300) as a mosaic and incubated for 24 hrs in a cell culture incubator at 37 °C, 5% CO _2_. Culture medium was removed, and cells were fixed with 4% paraformaldehyde (PFA) (VWR, cat. Number 100503–917) in phosphate buffered saline (PBS) (Wisent, cat. Number 311–010-CL) for 15 min at room temperature. Cells were permeabilized in PBS1x with 0.1% Triton X-100 (Thermo Fisher Scientific, cat. Number BP151–500) for 10 min at room temperature and blocked with PBS1x with 5% BSA, 5% goat serum (Gibco, cat. Number 16210–064) and 0.01% Triton X-100 for 30 min at room temperature. Cells were incubated with IF buffer (PBS, 5% BSA, 0.01% Triton X-100) containing the primary KIF5A antibodies overnight at 4 °C. Cells were then washed 3 × 10 min with IF buffer and incubated with the corresponding Fluor 555-conjugated secondary antibody in IF buffer for 1 hr at room temperature. Cells were washed 3 × 10 min with PBS1x then incubated with DAPI (Thermo Fisher Scientific, cat. Number D3571) and washed once with PBS.

Images were acquired on an ImageXpress micro confocal high-content microscopy system (Molecular Devices), using a 20x NA 0.94 air objective and scientific CMOS cameras, equipped with 395, 475, 555 and 635 nm solid state LED lights (lumencor Aura III light engine) and bandpass filters to excite DAPI, Cellmask Green, Alexa-555 and Cellmask Red, respectively. Images had pixel sizes of 0.68 x 0.68 microns, and a z-interval of 4 microns. For analysis and visualization, shading correction (shade only) was carried out for all images. Then, maximum intensity projections were generated using 3 z-slices. Segmentation was carried out separately on maximum intensity projections of Cellmask channels using CellPose 1.0, and masks were used to generate outlines and for intensity quantification. ^8^ Figures were assembled with Adobe Illustrator.

## Data Availability

Zenodo: Dataset for the KIF5A antibody screening study < https://doi.org/10.5281/zenodo.20534229>. Data are available under the terms of the Creative Commons Attribution 4.0 International license (CC-BY 4.0).
