# Supplementary material for: A guide to selecting high-performing renewable antibodies for KIF5A (Q12840) across western blot, immunoprecipitation, and immunofluorescence
Source: F1000Res. 2026 Jun 30;15:1030. [Version 1] doi: 10.12688/f1000research.184178.1 (PMC13563017; doi:10.12688/f1000research.184178.1)
Supplement: Peer review history

# Reviewer report: Reviewer response for version 1

**DOI:** 10.5256/f1000research.203297.r512728

**Published:** 2026-08-31

**Authors:** Deborah Moshinsky

**Affiliations:** 1 Institute for Protein Innovation, Boston, Massachusetts, USA

KIF5A is a member of the KIF5 family of kinesin motor proteins that mediates anterograde transport along microtubules, supporting axonal maintenance and function. The authors characterized 10 commercially available antibodies against this target for performance in Western Blot, immunoprecipitation, and immunofluorescence using standardized protocols as part of a larger initiative to provide data to aid researchers in selecting appropriate antibodies for their experiments. Sufficient details are given for the experiments to be reproduced. The authors do not provide data interpretation, but they do offer general guidance on interpretation.

 Regarding Table 4 (Figure 4?), the headings are mixed up in that under Western Blot, an IF cartoon is shown, and under Immunofluorescence, Western Blot is shown. Additionally, regarding the IF image in Table 4, the instance of staining in WT and KO cells is labeled 'Target Protein not detected' whereas a more accurate label would be 'Target protein not selectively detected'. This also applies to the example of the Western blot where there is a band for WT and KO (target protein not selectively detected).

Are sufficient details of methods and materials provided to allow replication by others?

Yes

Is the rationale for creating the dataset(s) clearly described?

Yes

Are the datasets clearly presented in a useable and accessible format?

Yes

Are the protocols appropriate and is the work technically sound?

Yes

Reviewer Expertise:

Antibody Characterization and Validation

I confirm that I have read this submission and believe that I have an appropriate level of expertise to confirm that it is of an acceptable scientific standard.

# Reviewer report: Reviewer response for version 1

**DOI:** 10.5256/f1000research.203297.r501651

**Published:** 2026-07-22

**Authors:** Karen Bowman

**Affiliations:** 1 University of Leicester, Leicester, England, UK

The study involved characterization of ten commercial antibodies, from five different companies, for the Kinesin Family Member 5A (KIF5A) protein, for use in western blot, immunoprecipitation, and immunofluorescence. The study formed part of a wider YCharOS collaboration to characterize commercial antibodies, which are renewable (recombinant and monoclonal), for human proteins using standardized protocols with the intention of improving antibody reproducibility issues. As a Data Note, it allows the direct comparison of the performance of commercially available antibodies to the KIF5A protein to aid scientists in choosing the most appropriate antibody for their studies. For example, in understanding how disease-related mutations affect the protein’s biochemical and cellular characteristics. Since KIF1A plays a critical role in the transport of mitochondria and synaptic vesicles from the soma to the synapse in neurons, this comparison would be of particular use in neuroscience, neurogenetics, and neurodegenerative disease research, and most importantly in amyotrophic lateral sclerosis (ALS) research.

 A committee of industry and academic representatives have endorsed the platform used, and the protocols were consistent with those in general use. The platform consisted of identification of a human cell line suitable for antibody characterization studies i.e., with adequate expression levels of the KIF5A protein to generate a measurable signal. Using the DepMap (Cancer Dependency Map Portal, RRID: SCR_017655) transcriptomics database, they found that the commercially available U-87 MG cell line expresses *KIF5A* transcript at a level above the average range for cancer cells analyzed, and therefore, was a logical choice for their study. The commercially available matched U-87 MG cell line with knockdown of the target gene was used to provide an appropriate negative control (U-87 MG *KIF5A* KO) and was also a logical choice. The final step was a series of antibody characterization procedures, limited to the most common research uses of antibodies. The interpretation of the results is left to the reader, with the study providing unbiased guidance. However, it gave some indication of the optimum dilutions of commercial antibodies. Limitations of the study are clearly stated.

 The U-87 MG cell line does not carry mutations in the *KIF5A *gene; therefore, it is assumed the use of wild type KIF5A did not affect antibody–epitope binding. It would be useful to know if any of the antibodies bound to mutant versions of KIF5A, for drug discovery purposes.

 The results suggest that during immunofluorescence studies, the reliability of the tested antibodies can be particularly variable, even when one of the vendors’ recommended applications. Some antibodies successfully bound to KIF5A even though immunofluorescence was not one of the vendor’s recommended applications.

Are sufficient details of methods and materials provided to allow replication by others?

Yes

Is the rationale for creating the dataset(s) clearly described?

Yes

Are the datasets clearly presented in a useable and accessible format?

Yes

Are the protocols appropriate and is the work technically sound?

Yes

Reviewer Expertise:

drug discovery and development.

I confirm that I have read this submission and believe that I have an appropriate level of expertise to confirm that it is of an acceptable scientific standard.
